# New Candidate Genes Affecting Rice Grain Appearance and Milling Quality Detected by Genome-Wide and Gene-Based Association Analyses

**DOI:** 10.3389/fpls.2016.01998

**Published:** 2017-01-04

**Authors:** Xiaoqian Wang, Yunlong Pang, Chunchao Wang, Kai Chen, Yajun Zhu, Congcong Shen, Jauhar Ali, Jianlong Xu, Zhikang Li

**Affiliations:** ^1^Institute of Crop Sciences/National Key Facility for Crop Gene Resources and Genetic Improvement, Chinese Academy of Agricultural SciencesBeijing, China; ^2^Agricultural Genomics Institute, Chinese Academy of Agricultural SciencesShenzhen, China; ^3^International Rice Research InstituteMetro Manila, Philippines; ^4^Shenzhen Institute of Breeding and Innovation, Chinese Academy of Agricultural SciencesShenzhen, China

**Keywords:** rice, GWAS, gene-based association analysis, grain shape, chalkiness, milling

## Abstract

Appearance and milling quality are two crucial properties of rice grains affecting its market acceptability. Understanding the genetic base of rice grain quality could considerably improve the high quality breeding. Here, we carried out an association analysis to identify QTL affecting nine rice grain appearance and milling quality traits using a diverse panel of 258 accessions selected from 3K Rice Genome Project and evaluated in two environments Sanya and Shenzhen. Genome-wide association analyses using 22,488 high quality SNPs identified 72 QTL affecting the nine traits. Combined gene-based association and haplotype analyses plus functional annotation allowed us to shortlist 19 candidate genes for seven important QTL regions affecting the grain quality traits, including two cloned genes (*GS3* and *TUD*), two fine mapped QTL (*qGRL7.1* and *qPGWC7*) and three newly identified QTL (*qGL3.4, qGW1.1*, and *qGW10.2*). The most likely candidate gene(s) for each important QTL were also discussed. This research demonstrated the superior power to shortlist candidate genes affecting complex phenotypes by the strategy of combined GWAS, gene-based association and haplotype analyses. The identified candidate genes provided valuable sources for future functional characterization and genetic improvement of rice appearance and milling quality.

## Introduction

As a major cereal crop, rice (*Oryza sativa* L.) is crucial to food security for more than half of the world's population. Rapid population growth coupled with the climate change creates an urgent need for rice varieties with high yield, high quality and stress tolerances. In the past half century, rice production has been significantly improved benefiting from the green revolution and the wide adoption of hybrid rice (Xu J. L. et al., 2015). However, for rice breeders and consumers, rice grain quality is also a foremost consideration which includes appearance, milling, cooking and eating, and nutritional quality. Grain appearance quality is a crucial factor affecting its market acceptability. Mainly, appearance quality indicates grain shape and chalkiness. Grain shape can be described by grain length (GL), grain width (GW), and grain length to width ratio (GLWR), which are closely associated with grain weight (Zheng et al., 2007; Qiu et al., 2015). Chalkiness is usually evaluated by the degree of endosperm chalkiness (DEC) and the percentage of grain with chalkiness (PGWC). Rice variety with PGWC more than 20% is not generally acceptable in most world markets (Chen et al., 2011). Milling quality is usually measured as brown rice rate (BRR), milled rice rate (MRR), and head-milled rice rate (HMRR).

Breeding rice varieties with desirable appearance and high milling quality is a paramount consideration for rice breeders. Understanding the genetic basis of these traits could considerably improve breeding efficiency. Rice grain appearance and milling related traits are quantitatively inherited and controlled by multiple genes/QTL (Tan et al., 2000). To date, many genes governing grain shape have been identified and cloned, such as *GW2* (Song et al., 2007), *GIF1* (Wang et al., 2008), *qSW5* (Shomura et al., 2008), *GS3* (Mao et al., 2010), *GS5* (Li et al., 2011), *qGL3* (Zhang et al., 2012), *GW8* (Wang S. et al., 2012), *GS6* (Sun et al., 2013), *GS2* (Hu et al., 2015), *GL7*/*GW7* (Wang S. et al., 2015; Wang Y. et al., 2015), *OsMAPK6* (Liu S. et al., 2015), and *GLW7* (Si et al., 2016). Besides these genes, many QTL affecting grain size have been identified through linkage mapping and association studies (Zhao et al., 2011; Singh et al., 2012; Zhang W. et al., 2013; Yang et al., 2014; Liu D. et al., 2015; Qiu et al., 2015; Edzesi et al., 2016; Feng et al., 2016), and some of them have been fine mapped such as *GW1-1* and *qGRL1.1* (Singh et al., 2012), *GW3* and *GW6* (Guo et al., 2009), *qGL-7* (Bai et al., 2010), *qGRL7.1* (Singh et al., 2012). For grain chalkiness, only one gene, *Chalk5* is cloned (Li et al., 2014). One QTL for PGWC, *qPGWC-7* was fine mapped to 44 kb region on chromosome 7 (Zhou et al., 2009), and one QTL cluster for chalkiness on chromosome 4 flanked by id4007289 and RM252 was detected by single environment analysis and joint mapping across nine environments (Zhao et al., 2016). No gene affecting milling quality is cloned. But recently, *qBRR-10* for BRR was narrowed to a 39.5 kb region on chromosome 10 and two candidate genes were determined (Ren et al., 2016).

The usefulness of some of the well characterized genes/QTL for grain shape and chalkiness is proven in an *Xian* (*indica*) population of diverse breeding lines (Zhao et al., 2015). Therefore, it's worthwhile to explore new genes/QTL regulating rice grain appearance and milling quality. Genome-wide association study (GWAS) of complex traits in rice has been successful promoted by the recent advances in high-throughput sequencing technologies. The high density SNP markers and gene annotation based on reference genome facilitate the rapid identification of candidate genes associated with interested traits. Recently, Yano et al. (2016) identified four new genes associated with agronomic traits in rice using GWAS and gene-based association analysis. The combination of GWAS and gene-based association analysis will accelerate the investigation of mechanism for rice quality.

In the present study, GWAS and gene-based association analysis were carried out to identify candidate genes associated with rice grain appearance and milling quality. A diverse panel consisting of 258 accessions selected from 3K Rice Genome Project (3K RGP) (3K RGP, 2014) was evaluated in two environments. GWAS was performed using 27K SNPs generated from 3K RGP through high-throughput sequencing technologies (Zheng et al., 2015). Then, for important QTL regions, gene-based association analysis was performed using all available SNP from Rice SNP-Seek Database (Alexandrov et al., 2015). By this way, a number of new candidate genes governing rice grain appearance traits were identified.

## Materials and methods

### Plant materials

To minimize the influence of flowering time on rice grain appearance and milling quality traits to be measured, we selected 258 rice accessions from the 3K RGP which have similar heading dates. These rice accessions are from 51 countries or regions and were used as the materials in this study. This panel consisted of seven types, including *Xian* (*indica*) (174), *temperate Geng (japonica)* (32), *tropical Geng (japonica)* (24), subtropical *Geng* (*japonica*) (14), *admixture* type (7), *aus*/*boro* (3), and *basmati*/*sadri* (4) (Supplementary Table S1).

### Field trials and trait measurements

All of these accessions were grown in two environments, including Sanya (18.3°N, 109.3°E) during Dec 2014–April, 2015 and Shenzhen (22.6°N, 114.1°E) during March–July, 2015. In both environments, each accession was planted in a two-row plot with 10 individuals planted in each row at a spacing of 20 cm × 25 cm with two replications for each accessions. The field management followed the local farmers' standard management practices. At maturity (about 40 days after flowering), eight uniform plants in the middle of each plot were bulk harvested and air-dried for 3 months in the drying houses. Then, around 150 g seeds were dehulled in an electrical dehuller (model JLGJ-45, China) and milled by a desk-top rice miller (JNMJ 6, China). Three traits related to grain milling quality were measured according to the National Rice Grain Quality Assessment Standard of China (GB/T17891-1999), including brown rice rate (BRR, %), milled rice rate (MRR, %) and head milled rice rate (HMRR, %). Then, all full head milled rice kernels of each accession were used to measure grain length (GL, mm), grain width (GW, mm), grain length-width ratio (GLWR), degree of endosperm chalkiness (DEC, %), percentage of grain with chalkiness (PGWC, %) and transparency (Tr) using a rice grain appearance quality scanning machine (SC-E, Wanshen Technology Company, Hangzhou, China). All measurements were conducted with samples of the two replications and the average trait value of each accession was used in data analyses of GWAS.

### Genotyping

The 27K SNP genotype data of the 258 accessions was generated from the 3K RGP (Zheng et al., 2015). For those SNPs with more than two alleles, only two alleles of highest frequency in the 258 panel were retained and other alleles of low frequency were considered missing. The heterozygous was also regarded as missing. SNP loci with missing rate over 20% and minor allele frequency (MAF) less than 0.05 were removed. Finally, a total of 22,488 SNPs were used in the GWAS.

### Population structure and kinship

For the 22,488 SNP, we further removed SNP loci with missing rate over 10% and MAF less than 0.1. Then, 8038 evenly distributed SNPs with average marker spacing around 50 kb were sampled to calculate population structure (Q) and kinship (K). For the population structure analysis, a model based Bayesian clustering analysis method implemented in STRUCTURE software version 2.3.4 (Pritchard et al., 2000) was used. The program was run with the following parameters: k, the number of groups in the panel varying from 1 to 10; 10 runs each k value; for each run, 10,000 burnin iterations followed by 10,000 MCMC (Markov Chain Monte Carlo) iterations. For K calculation, the default method, Centered_IBS, implemented in TASSEL 5.2.23 was utilized (Bradbury et al., 2007). The IBS was scaled to have the mean diagonal element equal to 1+F, where F is the inbreeding coefficient of the current population (Endelman and Jannink, 2012). The Q and K matrix were used in the following association analysis.

### Linkage disequilibrium (LD) analysis

LD was measured by squared allele frequency correlations (r^2^) values between the pairs of markers using 8038 SNP calculated by TASSEL 5.2.23 (Bradbury et al., 2007). Marker pairs were discretized into bins of 5 kb and the average r^2^ value was used as the estimate of r^2^ of a bin. The LD decay rate was measured as the chromosomal distance at which the average r^2^ dropped to half of its maximum value (Huang et al., 2010).

### GWAS and of candidate genes identification for QTL affecting measured traits

We performed a genome wide association study (GWAS) to detect the trait-SNP associations for all measured traits using 22,488 SNPs and the mean trait values of the 258 accessions from each of the environments. All statistical analyses for GWAS were performed using the SVS software package (SNP and Variation Suite, Version 8.4.0). An EMMAX (Efficient Mixed-Model Association eXpedited) (Kang et al., 2010; Vilhjalmsson and Nordborg, 2013) implementation of the single-locus mixed linear model was applied to the marker dataset. This mixed linear model allowed correction for cryptic relatedness and other fixed effects using a kinship matrix and population stratification using principle components. The Bonferroni multiple testing correction was applied to identify significant markers. A QTL affecting the measured traits were claimed when the test statistics reached *P* < 1.0 × 10^−4^ in at least one of the two environments.

Gene-based association analysis was carried out for to detect candidate genes for important QTL. Here, QTL regions meeting at least one of the following criteria were considered as important: (1) consistently identified in both environments; (2) affecting more than one trait; (3) accounting for over 10% of phenotypic variance, and/or (4) close to reported cloned genes or fine-mapped QTL. The following five steps were conducted to identify candidate genes for important QTL identified. We, firstly, found all the genes located in 0.31 LD block region of the peak SNP of each important QTL from the Rice Annotation Project Database (RAP-DB). Then, all available SNPs located inside of these genes were searched from 32 M SNPs data generated from 3K RGP in the Rice SNP-Seek Database (Alexandrov et al., 2015). The genotype manipulation was done in the same way as described above. Thirdly, the high quality SNPs inside of these candidate genes of each important QTL were used to perform gene-based association analyses through MLM using the Q and K applied in GWAS. For each QTL region, the SNPs whose –log10 (p) located in the interval of 1 unit of the maximum value were regarded as significant. Fourthly, haplotype analysis was carried out for each of the candidate genes in each important QTL region using all non-synonymous SNPs located inside of the gene CDS region. Finally, candidate genes were determined by testing the significant differences among major haplotypes (containing more than 10 samples) for each important QTL through ANOVA.

## Results

### Trait variance and correlations

**Figure 1:**
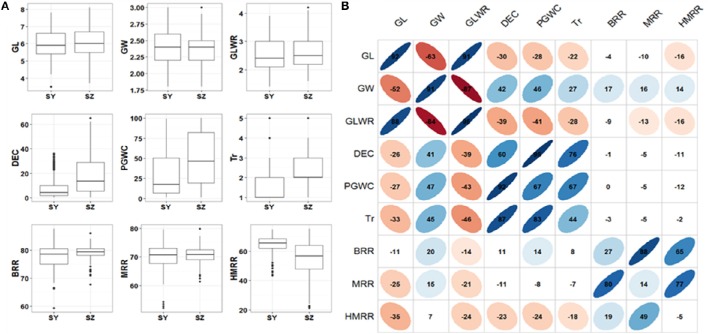
**(A)** Box plots of nine rice grain appearance and milling quality traits in two environments. SY, Sanya; SZ, Shenzhen; GL, Grain length; GW, Grain width; GLWR, Grain length to width ratio; DEC, Degree of endosperm chalkiness; PGWC, Percentage of grains with chalkiness; Tr, Transparency; BRR, Brown rice rate; MRR, Milled rice rate; HMRR, Head milled rice rate. **(B)** Correlations between nine evaluated traits in SY (upper triangular) and SZ (lower triangular). The values on principal diagonal indicated correlations between SY and SZ. The values were correlation coefficients (r) multiplied by 100. The areas and colors of ellipses showed the absolute value of corresponding r. Right and left oblique ellipses indicated positive and negative correlations, respectively. The values without glyphs indicated insignificant at 0.05.

In general, most of the traits appeared to be normally distributed, but some traits showed skewed distributions especially for Tr (Figure 1A). The panel showed a large variations for all the measured traits. Significant variations between SY and SZ were observed for DEC, PGWC, Tr, and HMRR, but not for other traits (Figure 1A). The phenotype pairwise correlations between the measured traits were similar in both environments. GL and GLWR were positively correlated with each other, and negatively correlated with GW. Positive correlations were observed between DEC, PGWC, and Tr, and they were negatively correlated with GL and GLWR, but positively correlated with GW. Overall, the correlations between appearance quality and milling quality traits were very weak. The three milling traits BRR, MRR and HMRR showed positive correlations with one another, but their correlations between two environments were very poor (Figure 1B).

### Basic statistics of markers

For the 22,488 high quality SNPs data, the number of markers per chromosome ranged from 1360 on chromosome 9–2783 on chromosome 1. The size of chromosome varied from 22.9 Mb for chromosome 9 to 43.2 Mb for chromosome 1. The whole genome size was 372.2 Mb. The average marker spacing was 16.6 kb with spacing ranging from 15.3 kb for chromosomes 8 and 10–18.7 kb for chromosome 7 (Table 1). More than half (57.4%) of the markers had MAF more than 0.20 (Supplementary Figure S1).

**Table 1:** **Distributions of markers on chromosomes**.

**Chr**	**Marker no**.	**Size (Mb)**	**Spacing (kb)**
Chr1	2783	43.2	15.5
Chr2	2268	35.9	15.8
Chr3	2086	36.3	17.4
Chr4	1964	35.5	18.1
Chr5	1740	29.7	17.1
Chr6	1911	31.1	16.3
Chr7	1583	29.7	18.7
Chr8	1864	28.4	15.3
Chr9	1360	22.9	16.8
Chr10	1510	23.1	15.3
Chr11	1869	29.0	15.5
Chr12	1550	27.4	17.7
Total	22,488	372.2	16.6

### Population structure and LD patterns

**Figure 2:**
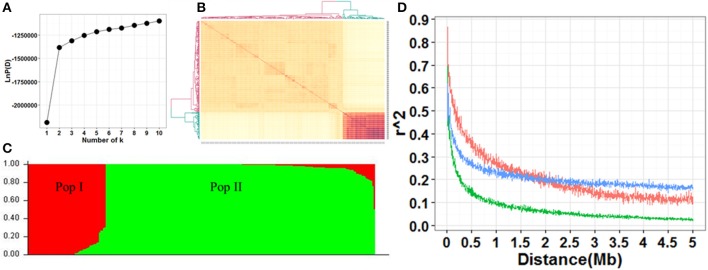
**(A)** Screen plot from STRUCTURE showing the selection of Q for association study. **(B)** Heat map of kinship from TASSEL with the tree shown on the top and left. **(C)** Bayesian clustering of 258 accessions using STRUCTURE program. **(D)** Comparison of LD decay in the whole and two sub-populations. Y axis was the average r^2^ value of each 5 kb region and X axis was physical distance between markers in unit of Mb. The blue, red and green indicated LD decay in the populations whole, Ppo I and Pop II, respectively.

The screen plot generated through STRUCTURE recommended *k* = 2 as informative, where ascent changed gradually (Figure 2A). There were two distinct subpopulations (Pop I and Pop II) in the current panel according to the results of STRUCTURE and kinship (Figures 2A,B). Pop I consisted of 58 accessions, most of which were *temperate Geng* (22), *tropica Geng* (16) and *subtropical Geng* (9). Pop II consisted of 200 accessions, most of which were *Xian* (167). In this panel, 53% (136/258) of the accessions did not show any admixture and 37% (96/258) showed less than 10% admixture, while the remaining 10% (26/258) were found to be highly admixed (Figure 2C). Overall, the LD decay in Pop II was much faster than Pop I. The maximum LD was 0.62, 0.87, and 0.70 in the whole population, Pop I and Pop II, respectively. LD reached half of its initial value at around 100 kb in Pop II, and 300 kb in Pop I and the whole population (Figure 2D).

### Detection of QTL by GWAS

A total of 72 QTL for all investigated traits were identified in SY and SZ, ranging from two QTL for HMRR to as many as 18 QTL for GW. Among them, 21 (36) QTL were detected only in SY (SZ), and 15 QTL were commonly identified in both two environments (Table 2).

**Table 2:** **QTL identified for nine traits in two environments**.

**QTL**	**Env**	**Peak SNP**	**Alleles^a^**	**MAF**	***p***	**Effect^b^**	***R*^2^(%)^c^**
*qGL2*	SZ	S2_7684141	C/T	0.12	2.5E-05	−0.59	8.2
*qGL3.1*	SY	S3_1249933	T/C	0.13	1.7E-05	−0.52	7.8
	SZ	S3_1249933	T/C	0.14	7.2E-05	−0.49	7.2
*qGL3.2*	SY	S3_5043816	G/A	0.11	6.1E-05	0.74	6.8
*qGL3.3*	SY	S3_15049416	A/G	0.12	3.4E-05	−0.80	7.2
	SZ	S3_15434503	C/T	0.12	5.1E-06	−0.90	9.5
*qGL3.4*	SY	S3_15745213	C/T	0.13	3.0E-06	−0.77	9.2
	SZ	S3_15745213	C/T	0.13	3.0E-05	−0.71	8.1
*qGL3.5*	SY	S3_16883926	G/A	0.49	2.4E-06	0.66	9.6
	SZ	S3_16785761	A/G	0.47	2.3E-05	−0.61	8.5
*qGL4.1*	SZ	S4_17471146	T/C	0.35	3.1E-05	0.45	8.5
*qGL4.2*	SZ	S4_18248441	T/C	0.12	5.2E-05	0.63	7.5
*qGL4.3*	SY	S4_20297417	G/A	0.15	2.8E-05	0.74	8.0
	SZ	S4_20137296	T/A	0.08	8.7E-05	0.61	7.0
*qGL6.1*	SY	S6_3562109	C/T	0.13	9.2E-05	0.49	6.5
*qGL6.2*	SZ	S6_8736179	C/T	0.09	3.5E-05	−0.67	7.7
*qGL7*	SY	S7_22569856	A/T	0.20	1.7E-05	−0.62	7.8
	SZ	S7_22569856	A/T	0.22	1.0E-04	−0.57	6.6
*qGL8*	SZ	S8_2849446	A/T	0.11	3.6E-06	−0.67	11.1
*qGL9*	SZ	S9_8688246	C/T	0.32	8.1E-06	−0.49	9.5
*qGW1.1*	SY	S1_10935666	C/A	0.13	3.8E-05	−0.20	7.2
	SZ	S1_10935666	C/A	0.13	3.2E-05	−0.19	8.0
*qGW1.2*	SZ	S1_35120676	G/A	0.18	1.1E-05	−0.30	8.9
*qGW3.1*	SZ	S3_7012384	T/C	0.21	5.7E-05	−0.51	7.4
*qGW3.2*	SY	S3_15203521	G/A	0.16	2.7E-05	0.22	7.4
	SZ	S3_15049416	A/G	0.14	3.7E-05	0.29	7.7
*qGW4.1*	SZ	S4_20331973	T/C	0.06	4.8E-05	0.29	7.6
*qGW4.2*	SZ	S4_31788543	G/A	0.04	9.2E-05	0.31	7.1
*qGW5*	SY	S5_5369802	G/A	0.36	8.9E-07	0.17	10.4
	SZ	S5_5459847	A/G	0.32	1.0E-04	0.14	6.5
*qGW6*	SZ	S6_24696098	C/T	0.07	9.2E-05	0.19	7.3
*qGW7.1*	SZ	S7_19709162	G/A	0.10	5.2E-05	−0.24	7.8
*qGW7.2*	SY	S7_20971202	C/T	0.07	3.5E-05	−0.29	7.8
*qGW7.3*	SY	S7_22569856	A/T	0.20	6.4E-05	0.21	6.7
*qGW7.4*	SY	S7_23080276	G/T	0.13	6.2E-05	0.19	6.7
	SZ	S7_22980051	G/A	0.06	2.6E-05	−0.34	8.1
*qGW8.1*	SZ	S8_21536949	G/A	0.18	6.8E-05	0.34	7.5
*qGW8.2*	SZ	S8_27055234	G/A	0.19	7.0E-06	−0.29	9.5
*qGW9*	SZ	S9_20389437	A/T	0.06	9.2E-05	0.21	7.1
*qGW10.1*	SZ	S10_13811940	C/T	0.09	8.2E-05	0.22	7.2
*qGW10.2*	SY	S10_19624722	T/C	0.25	1.7E-05	0.20	7.8
	SZ	S10_19624722	T/C	0.27	1.6E-05	0.21	8.5
*qGW11*	SZ	S11_5752053	C/A	0.16	1.3E-05	0.19	8.8
*qGLWR1*	SZ	S1_2698492	C/T	0.23	9.5E-05	0.29	7.6
*qGLWR2*	SY	S2_34856918	C/T	0.14	8.7E-05	0.42	6.4
*qGLWR3.1*	SZ	S3_14988992	C/A	0.12	2.1E-06	−0.66	10.3
*qGLWR3.2*	SY	S3_16785761	A/G	0.47	9.9E-05	−0.37	6.4
	SZ	S3_16785761	A/G	0.47	5.6E-05	−0.38	7.5
*qGLWR4.1*	SZ	S4_17267620	G/T	0.08	8.1E-05	0.37	7.3
*qGLWR4.2*	SZ	S4_18248441	T/C	0.12	6.6E-05	0.41	7.3
*qGLWR5*	SY	S5_5369802	G/A	0.36	1.8E-05	−0.31	7.8
	SZ	S5_5369802	G/A	0.35	9.0E-05	−0.29	6.9
*qGLWR7*	SY	S7_22569856	A/T	0.20	9.0E-05	−0.49	9.7
	SZ	S7_22569856	A/T	0.22	1.2E-05	−0.44	8.7
*qGLWR9*	SZ	S9_8714326	G/A	0.11	2.7E-05	0.40	8.0
*qGLWR10*	SZ	S10_19552708	T/C	0.38	6.3E-05	−0.32	7.8
*qGLWR11.1*	SZ	S11_5752053	C/A	0.16	3.8E-05	−0.32	7.8
*qGLWR11.2*	SY	S11_17039961	C/G	0.23	4.8E-05	−0.55	6.9
*qGLWR12*	SZ	S12_26246050	C/T	0.06	3.8E-05	0.42	7.6
*qDEC1*	SZ	S1_5420348	G/A	0.23	7.3E-05	−29.8	7.3
*qDEC3*	SZ	S3_7302378	C/T	0.10	8.7E-06	14.7	9.3
*qDEC7*	SY	S7_24749850	C/T	0.05	1.1E-05	12.3	8.2
*qDEC8*	SZ	S8_27055234	G/A	0.19	6.2E-05	−16.9	7.5
*qPGWC3*	SZ	S3_7489318	C/T	0.20	1.2E-05	−48.2	8.9
*qPGWC5*	SY	S5_5369802	G/A	0.36	1.9E-05	19.6	7.9
	SZ	S5_5369802	G/A	0.35	9.3E-06	23.2	9.6
*qPGWC8*	SZ	S8_27055234	G/A	0.19	2.2E-05	−39.5	8.4
*qPGWC10*	SZ	S10_13811940	C/T	0.09	7.4E-05	31.4	7.4
*qTr1*	SY	S1_29263696	A/T	0.06	1.7E-05	1.4	7.8
*qTr2*	SZ	S2_18924283	C/T	0.30	3.4E-05	1.1	8.1
*qTr4*	SY	S4_923547	C/A	0.23	5.6E-05	0.8	8.8
*qTr7.1*	SY	S7_6134470	A/C	0.05	1.2E-06	1.4	10.6
*qTr7.2*	SY	S7_24749850	C/T	0.05	4.5E-05	1.2	7.0
*qTr7.3*	SY	S7_25428951	T/C	0.29	9.9E-05	1.2	6.5
*qBRR1.1*	SZ	S1_40365293	T/A	0.23	2.8E-05	−2.8	7.9
*qBRR1.2*	SZ	S1_42736913	C/T	0.23	7.5E-05	−1.6	7.1
*qBRR3*	SY	S3_34735871	G/A	0.08	5.4E-05	−3.9	6.9
*qBRR7*	SZ	S7_8452759	C/T	0.06	5.3E-05	−2.9	7.3
*qBRR9*	SY	S9_9144846	T/C	0.28	3.8E-05	−3.7	7.2
*qBRR11*	SY	S11_23855546	C/T	0.25	2.9E-06	3.0	9.3
*qMRR3*	SY	S3_34735871	G/A	0.08	5.2E-07	−5.0	11.1
*qMRR9*	SY	S9_10381563	G/C	0.24	3.6E-05	−5.6	7.5
*qMRR10*	SY	S10_15603572	A/T	0.20	5.8E-05	6.8	6.8
*qMRR11.1*	SY	S11_23855546	C/T	0.25	2.0E-05	2.9	8.1
*qMRR11.2*	SY	S11_27415368	G/A	0.19	7.2E-05	−2.8	6.7
*qHMRR3*	SZ	S3_15745213	C/T	0.14	7.9E-05	13.4	7.1
*qHMRR9*	SY	S9_10381563	G/C	0.24	8.4E-05	−7.4	6.8

a *Major/Minor allele*.

b *Effect: Allele effect with respect to the minor allele*.

c *R^2^ (%): Phenotypic variance explained*.

For GL, 14 QTL were detected on chromosomes 2–4 and 6–9. Two QTL, *qGL3.2* and *qGL6.1* were detected only in SY and explained 6.8 and 6.5% of phenotypic variance, respectively. Six QTL were detected only in SZ including *qGL2, qGL4.1, qGL4.2, qGL6.2, qGL8*, and *qGL9*, and accounted for 7.5 to 11.1% of phenotypic variance. Six QTL including *qGL3.1, qGL3.3, qGL3.4, qGL3.5, qGL4.3*, and *qGL7* were detected in both environments and the phenotypic variance explained ranged from 7.2 (6.6) to 9.6% (9.5%) in SY (SZ) (Table 2).

Eighteen QTL for GW were detected on all chromosomes except 2 and 12. Two QTL, *qGW7.2*, and *qGW7.3*, were detected only in SY, and accounted for 7.8 and 6.7% of phenotypic variance, respectively. Eleven QTL were detected only in SZ including *qGW1.2, qGW3.1, qGW4.1, qGW4.2, qGW6, qGW7.1, qGW8.1, qGW8.2, qGW9, qGW10.1*, and *qGW11* with phenotypic variance explained ranging from 7.1 to 9.5%. Five QTL were identified in both SY and SZ including *qGW1.1, qGW3.2, qGW5, qGW7.4*, and *qGW10.2* with phenotypic variance accounted for ranging from 6.7 (6.5) to 10.4% (8.5%) in SY (SZ) (Table 2).

For GLWR, 13 QTL were detected on all chromosomes except 6 and 8. Two QTL, *qGLWR2*, and *qGLWR11.2* were detected only in SY and accounted for 6.4 and 6.9% of phenotypic variance, respectively. Eight QTL were detected only in SZ including *qGLWR1, qGLWR3.1, qGLWR4.1, qGLWR4.2, qGLWR9, qGLWR10, qGLWR11.1*, and *qGLWR12* with phenotypic variance explained ranging from 7.3 to 10.3%. Three QTL, *qGLWR3.2, qGLWR5*, and *qGLWR7*, were identified in both environments and accounted for 6.4 (7.5), 7.8 (6.9), 9.7% (8.7%) of phenotypic variance in SY (SZ), respectively (Table 2).

Four QTL affecting DEC were detected on chromosomes 1, 3, 7, and 8. One QTL, *qDEC7*, was identified only in SY and explained 8.2% of phenotypic variance. The other three QTL, *qDEC1, qDEC3*, and *qDEC8*, were detected only in SZ with phenotypic variance accounted for being 7.3, 9.3, and 7.5%, respectively. For PGWC, four QTL were identified on chromosomes 3, 5, 8, 10. Three QTL were detected only in SZ including *qPGWC3, qPGWC8*, and *qPGWC10* and explained 8.9, 8.4, and 7.4% of phenotypic variance, respectively. One QTL, *qPGWC5*, were detected in both of SY and SZ accounting for 7.9 and 9.6% of phenotypic variance, respectively. Six QTL for Tr were detected on chromosomes 1, 2, 4, and 7. Five QTL were detected only in SY including *qTr1, qTr4, qTr7.1, qTr7.2* with phenotypic variance explained ranging from 6.5 to 10.6%. One QTL, *qTr2*, was identified only in SZ accounting for 8.1% of phenotypic variance (Table 2).

For BBR, six QTL were detected on chromosomes 1, 3, 7, 9, and 11. Three QTL, *qBRR3, qBRR9*, and *qBRR11*, were identified only in SY, and explained 6.9, 7.2, and 9.3% of phenotypic variance, respectively. Three QTL were detected only in SZ including *qBRR1.1, qBRR1.2*, and *qBRR7* accounting for 7.9, 7.1, and 7.3% of phenotypic variance, respectively. Five QTL (*qMRR3, qMRR9, qMRR10, qMRR11.1*, and *qMRR11.2*) for MRR were identified on chromosomes 3, 9, 10, and 11 only in SY, and the phenotypic variance accounted for ranged from 6.7 to 11.1%. Two QTL affecting HMRR, were identified on chromosomes 3 and 9. One QTL, *qHMRR9* was identified in SY and explained 6.8% of phenotypic variance. The other QTL, *qHMRR3*, was identified in SZ and accounted for 7.1% of phenotypic variance (Table 2).

### Candidate genes for important QTL

Supplementary Table S2 shows the list of 19 candidate genes shortlisted for seven important QTL regions based on the haplotype analyses of non-synonymous SNPs within each of the genes locating inside 0.31 LD decay of the peak SNPs, ranging from one to five candidate genes for each region.

**Figure 3:**
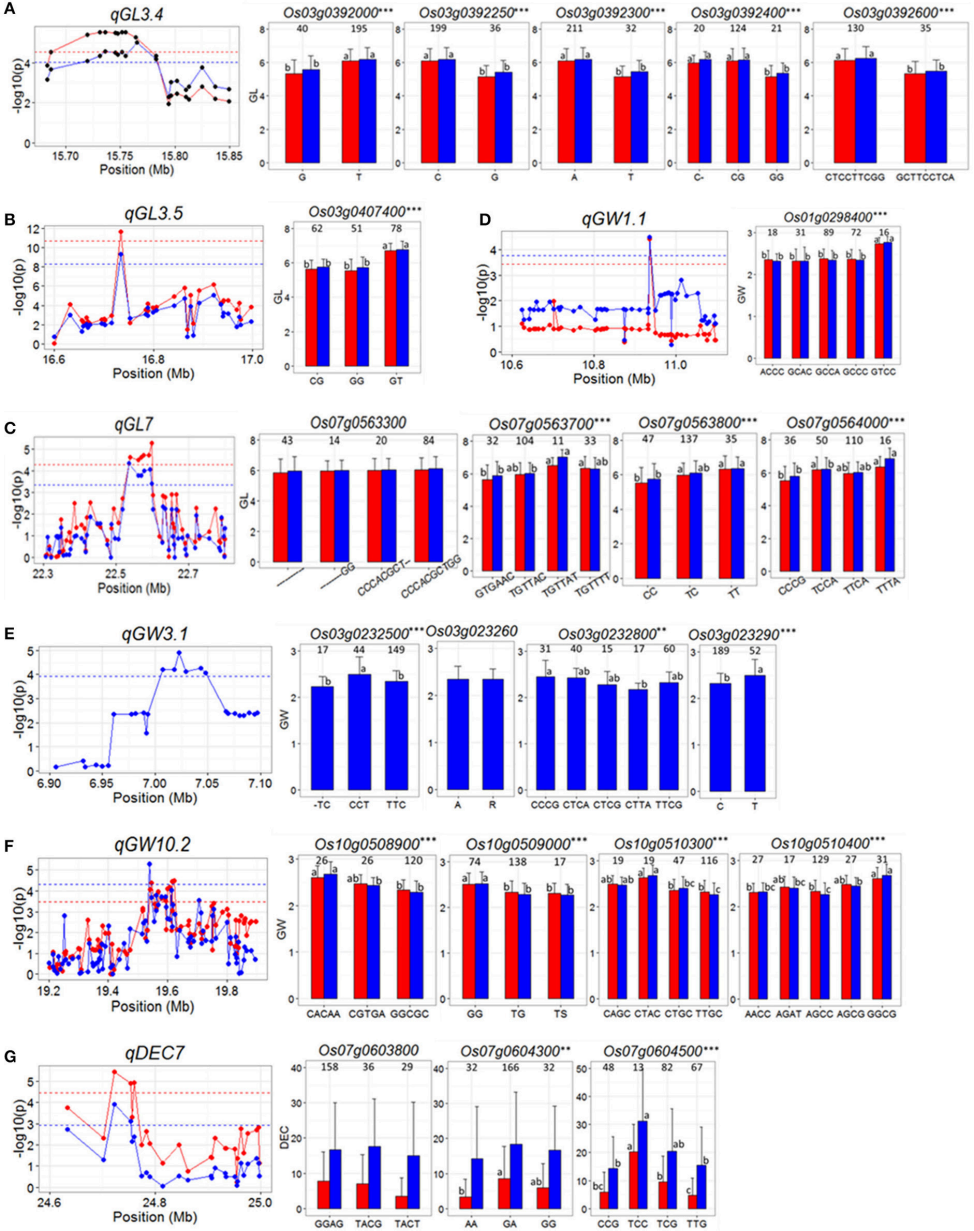
**(A–G)** Gene-based association analysis of seven important QTL loci and haplotypes analysis of targeted genes of related QTL including *qGL3.4* **(A)**, *qGL3.5* **(B)** *qGL7* **(C)**, *qGW1.1* **(D)**, *qGW3.1* **(E)**, *qGW10.2* **(F)**, and *qDEC7* **(G)**. Each point was a gene indicated by one of its SNPs having largest LD (r^2^) value with the peak SNP of the QTL. Dash line showed the threshold to determine significant SNP. The ^**^ and ^***^ suggested significance of ANOVA at *p* < 0.01 and *p* < 0.001, respectively. The letter on histogram (a, b, and c) indicated multiple comparisons result at the significant level 0.01. The value on the histogram was the number of individuals of each haplotype. Red and blue color indicated SY and SZ environments, respectively.

For *qGL3.4* in the region of 15.68–15.85 Mb on chromosome 3, 503 SNPs in 20 genes were used for association analysis and then it was narrowed down a ~100 kb region containing eight genes, *Os03g0391850, Os03g0392000, Os03g0392050, Os03g0392200, Os03g0392250, Os03g0392300*, and *Os03g0392600* (Figure 3A). Highly significant differences in GL were detected between different haplotypes at five candidate genes (*Os03g0392000, Os03g0392250, Os03g0392300, Os03g0392400*, and *Os03g0392600*), and in all the five cases, significantly reduced GL was associated with the minor allele(s) (Figure 3A and Supplementary Table S2), as originally detected in the peak SNP (Table 2). Of the five genes, *Os03g0392400* was less likely the candidate since a single cytosine deletion within it that causes a frame shift mutation showed the same GL phenotype as haplotype CG causing a non-synonymous mutation.

In the region from 16.6 to 17.0 Mb on chromosome 3 harboring *qGL3.5* on chromosome 3, 5046 SNPs of 33 genes were used for association analysis. *qGL3.5* was fined mapped into a 35 kb region containing a single cloned gene, *Os03g0407400* (*GS3*) (Mao et al., 2010; Figure 3B). Three major haplotypes of *GS3* were found. Haplotype GT was associated with significantly longer GL than haplotypes CG and GG (Figure 3B and Supplementary Table S2).

For *qGL7*, in the region of 22.3 to 22.8 Mb on chromosome 7, 1059 SNPs in 74 genes were used for association analysis, which narrowed *qGL7* down to a ~80 kb region containing six genes, *Os07g0563300, Os07g0563700, Os07g0563800, Os07g0564000, Os07g0564100*, and *Os07g0564150* (Figure 3C). Three haplotypes were found for *Os07g0563800*, and four haplotypes were found for the other five genes. Significant differences for GL among haplotypes of all genes were observed except for that of *Os07g0563300* (Figure 3C and Supplementary Table S2).

For *qGW1.1*, in the region of 10.6–11.1 Mb on chromosome 1, 1068 SNP of 52 genes were used for association analysis and then it was fined mapped into ~25 kb region containing a single gene, *Os01g0298400* (Figure 3D). Five major haplotypes were observed for *Os01g0298400*. Haplotype GTCC showed significantly wider GW than the other four haplotypes. The peak SNP of *qGW1.1* in Table 2 was just the fourth SNP of haplotype of *Os01g0298400* (Figure 3D and Supplementary Table S2).

For *qGW3.1*, in the region of 6.9–7.1 Mb on chromosome 3, 466 SNPs of 26 genes were used for association analysis, which narrowed *qGW3.1* down to a ~40 kb region containing six genes, *Os03g0232301, Os03g0232400, Os03g0232500, Os03g0232600, Os03g0232800, Os03g0232900* (Figure 3E). No haplotype was found in *Os03g0232301* and *Os03g0232400*. Three, five and two haplotypes were found for *Os03g0232500, Os03g0232800*, and *Os03g0232900*. The difference of GW between two haplotypes of *Os03g0232600* was insignificant. Significant differences in GW between haplotypes of the other three genes were observed (Figure 3E and Supplementary Table S2). But for *Os03g0232900*, reduced GW was associated with the major allele, which was inconsistent with detected in peak SNP (Table 2). These results indicated that *Os03g0232500* and *Os03g0232800* are the candidate genes for *qGW3.1*.

For *qGW10.2*, in the region of 19.2–19.9 Mb on chromosome 10, 2819 SNPs of 92 genes used for association analysis and then it was narrowed down to a ~100 kb region (Figure 3F) containing four genes *Os10g0508900, Os10g0509000, Os10g0510300*, and *Os10g0510400*. Haplotypes analysis revealed significant differences for GW were between different haplotypes at each of the four genes (Figure 3F and Supplementary Table S2), indicating that they are the candidate genes for *qGW10.2*.

*qDEC7* was detected in the region from 24.6 to 25.0 Mb on chromosome 7 where harboring 976 SNPs of 26 genes. Gene-based analysis using these SNPs narrowed *qDEC7* down into a ~70 kb region, in which three genes were harboring significant SNPs (Figure 3G). Haplotype analysis suggested that only the haplotypes of *Os07g0604500* showed significant differences in DEC in both environments (Figure 3G and Supplementary Table S2).

## Discussion

### Influences of population structure and LD decay on GWAS

The panel used in this study consisted of two populations, representing the two major subspecies of rice, *Xian* (*indica*, Pop II) and *Geng* (*japonica*, Pop I) (Figures 2A–C), which are known to differ greatly for the grain quality traits investigated in this study. Thus, most QTL identified in the panel are those loci contributing to the subspecific differences. We observed the LD decay in *Xian* accessions was approximately three times as fast as that in *Geng* accessions (Figure 2D). This strikingly difference in LD decay between the two major subspecies was expected from their difference in outcrossing rate (*Xian* accessions have much higher outcrossing rate than *Geng* accessions), from their distinct geographic distributions, and from their largely independent evolutionary (breeding) histories. In fact, the same results were also reported in previous researches (Huang et al., 2010; Zhao et al., 2011). Although LD decay distance is an important factor in determining the association mapping resolution (Flint-Garcia et al., 2003), the average marker density of 16.6 kb for the 22,488 SNPs used for GWAS was much smaller than the highest LD decay of ~300 kb in *Geng* accessions. In other words, the 22,488 SNPs were enough to capture most, if not all, marker-trait associations in the panel. Furthermore, the 0.31 LD block region of the peak SNP for each QTL was large enough to contain the targeted gene of related QTL. Therefore, application of gene-based association analysis using saturated SNPs in the 0.31 LD region flanking peak SNPs was reasonable to identify candidate genes.

### Candidate gene identification of the important QTL

Cloning QTL affecting complex traits has been a major challenge to plant geneticists and molecular biologists since the classical strategy using map-based cloning for QTL cloning is extremely troublesome and time-consuming. Using GWAS and gene-based association analysis combining with haplotype analysis of candidate genes, we were able to shortlist 19 candidate genes governing 7 important QTL affecting the measured traits. These candidates included two cloned QTL genes governing grain size. The first one was *qGL3.5* (*qGLWR3.2*), for which the results pinpointed a single candidate, *GS3* (*Os03g0407400*) functioning as a negative regulator for grain length (Fan et al., 2006). A nonsense mutation in the second exon of *GS3* causing 178-aa truncation in the C-terminus of the protein was identified in most large-grain varieties. The second one was *qGW3.1*, for which five candidate genes were identified. One of these genes was *TUD1* (*Os03g0232600*) encoding a U-Box E3 ubiquitin ligase. *TUD1* directly interacts with D1 mediating a BR-signaling pathway to affect plant growth and development including grain size (Hu et al., 2013).

Besides above two cloned genes, two previously fine-mapped QTL were also identified. In the region of 22.3–22.8 Mb on chromosome 7, a QTL cluster (*qGL7, qGLWR7*, and *qGW7.3*) was detected in the region of a fine mapped QTL (*qGRL7.1*) affecting GL, GW, and GLWR (Singh et al., 2012). Our haplotype analysis suggested five candidates for this QTL, including *Os07g0563700* (IKI3 family protein), *Os07g0563800* (a GTPase-activating protein), *Os07g0564000* (Conserved hypothetical protein), *Os07g0564100* (a UDP-glucuronosyl / UDP-glucosyltransferase family protein) and *Os07g0564150* (a hypothetical gene). In the chromosome region of 24.6–25.0 Mb, *qDEC7* (*qTr7.2*) were detected. This region harbors a fine mapped QTL *qPGWC7* flanked by InDel 14 and InDel 3 (Zhou et al., 2009). One candidate gene was determined in our analyses, *Os07g0604500* (mitochondrial import inner membrane translocase subunit Tim17).

Our results suggest five candidate genes for *qGL3.4*, a single candidate gene for *qGW1.1* and four candidates for *qGW10.2*. Of the five candidate genes for *qGL3.4*, the most likely one was *Os03g0392600* (OsSCP14, a putative serine carboxypeptidase homolog) because a cloned QTL gene, *GS5*, that positively regulates grain size, also encodes an OsSCP26, putative serine carboxypeptidase (Li et al., 2011; Xu C. et al., 2015). Another likely candidate gene for *qGL3.4* was *Os03g0392300*, a putative ADP-ribosylation factor (ARF) belonging to Ras superfamily of small GTP-binding proteins (GTPases) (Muthamilarasan et al., 2016). Overexpression of maize ARFs (*ZmARF1* and *ZmARF2*) in Arabidopsis could increase seed size (Wang et al., 2016). The only candidate gene for *qGW1.1, Os01g0298400* encodes a MYB family transcription factor. The MYB family transcription factors include many member genes with diverse functions in various biological processes including primary and secondary metabolism, plant development, cell fate and identity, and responses to biotic and abiotic stresses in all eukaryotes (Dubos et al., 2010). Some MYBs are known to be involved in regulating seed size in Arabidopsis and maize (Gupta et al., 2006; Zhang Y. et al., 2013). Thus, it's possible that the *Os01g0298400* may affect grain size in rice. Of the four candidate genes for *qGW10.2, Os10g0510300* encodes a putative ubiquitin carboxyl-terminal hydrolase 1 domain containing protein. It belongs to deubiquitinating enzyme that plays an important role in ubiquitination process. Ubiquitin carboxyl-terminal hydrolase 1 also has functions of ubiquitin ligase (Wing, 2003). Previous researches found *GW2* (*Os02g0244100*) governing GW and grain weight in rice encodes RING-type E3 ubiquitin ligase (Song et al., 2007). *GW2* negatively regulates cell division by targeting its substrate(s) to proteasomes for regulated proteolysis. Therefore, *Os10g0510300* is considered as the most likely candidate gene of *qGW10.2*. Transgenic experiments are under way to verify the functionalities of above candidate genes.

### Limitations of gene-based association analysis

Phenotypic variation is usually caused by non-synonymous mutations inside of genes, such as *GS3, GIF1, qGL3, GS2, GS6*, and *GLW7*, therefore, using SNPs inside of genes to detect candidate genes associated with investigated traits is logically applicable. However, polymorphisms in promoter regions of genes also induce phenotypic diversity, such as *Chalk5, GS5*, and *GW7*. These genes cannot be detected by the method applied in the present study. This problem could be partially solved by combining association analysis with expression profiling data (Yano et al., 2016).

We utilized gene models in the Nipponbare reference genome to perform gene-based association analysis. The genes that are missing in Nipponbare can't be identified. This was particularly true in this study as discussed above that we were primarily detecting loci contributing to the subspecific differences in the measured traits. For instance, we identified a QTL at the region of 5.3–5.5 Mb on chromosome 5 affecting GW, GLWR, and PGWC. A known gene *qSW5* governing GW that was deleted in Nipponbare was also located in this region. There is no gene locus ID of *qSW5* in RAP-DB, so *qSW5* cannot be detected through gene-based association analysis. Now, more high quality rice reference genomes are available (Zhang et al., 2016), which will help to solve this problem.

### Application in rice breeding for improved grain quality

In this study, GW was positively correlated with chalkiness traits including DEC, PGWC and Tr (Figure 1B). The positive correlations of GW with chalkiness were also reported in previous studies (Li et al., 2014; Qiu et al., 2015; Zhao et al., 2015; Zhou et al., 2015). This phenomenon could be partially explained by tightly linked QTL or QTL pleiotropy for GW and chalkiness. Qiu et al. (2015) identified a QTL region at 5.3 Mb on chromosome 5 affecting GW, DEC and PGWC with the same directions of allele effects. Li et al. (2014) reported that the tightly linkage of *Chalk5, GS5* and *qSW5* induced the unfavorable association of grain width and chalkiness. In the present study, three QTL affecting both GW and chalkiness were identified. They were *qGW5* and *qPGWC5, qGW8.2, qDEC8*, and *qPGWC8*, and *qGW10.1* and *qPGWC10*. The allele effects on GW and chalkiness traits were consistent at each QTL region (Table 2). Even so, three *Xian* accessions, IRIS_313.10430, IRIS_313.8087, and IRIS_313.8164 with wide GW but low chalkiness were found in this panel (Supplementary Table S3). At the three QTL regions mentioned above, these lines had the alleles reducing GW and chalkiness while they had the alleles increasing GW at six, six and nine of the other 15 GW QTL (Supplementary Table S3). At the other nine QTL for chalkiness traits, these lines had the alleles all decreasing chalkiness except at *qTr2* and *qTr7.3* (Supplementary Table S3). Therefore, improved grain quality with wide GW and low chalkiness of these three lines could be attributed to appropriate combinations of above alleles at different QTL for GW and chalkiness. Thus, these accessions could be used as favorable donors in *Geng* rice breeding for improved grain quality with wide grain and low chalkiness.

Meanwhile, we observed a general negative correlation between GL and GW, which was apparently due to opposite gene effects at the detected QTL for the two traits. Unexpectedly, QTL that increase both GL and GW was previously reported by Xu et al. (2004). In the present study, three QTL regions affecting both GL and GW were identified, and two QTL regions (*qGL3.3*/*qGW3.2*, and *qGL7*/*qGW7.3*) had opposite directions of allele effects on GL and GW (Table 2). But for the region of 20.2–20.4 Mb on chromosome 4 containing *qGL4.3* and *qGW4.1*, the allele effects on GL and GW were in the same direction in the two environments. So, This QTL region (*qGL4.3* and *qGW4.1*) could be a target in rice breeding to simultaneously increase GL and GW. Actually, six, five, three, and one QTL for GL, GW, GLWR, and PGWC, were shared between SY and SZ, respectively (Table 2). These environmental stable QTL could be utilized for improving grain shape or appearance quality by MAS in both two environments.

## Conclusion

Considerable genetic variations for nine grain quality traits existed in the panel consisting of 258 accessions of two major subspecies. Through GWAS, a total of 72 QTL for all investigated traits were identified. A total of 19 candidate genes of seven important QTL regions were determined by gene-based association and haplotype analyses, including two known genes *GS3* and *TUD*, and two previously fine mapped QTL *qGRL7.1* and *qPGWC7*. Four most likely candidates of three new QTL loci (*qGL3.4, qGW1.1*, and *qGW10.2*) governing grain size were inferred according to functional annotation. These candidate genes of new loci affecting rice grain appearance and milling quality provide valuable information for future functional characterization and MAS-based breeding for improving rice grain quality.

## Author contributions

ZL and JX designed the experiment; XW, KC, YZ, and CS performed all the phenotypic evaluation; YP and CW performed analysis and interpretation of the data; XW, YP, and JX drafted the manuscript; ZL and YP revised the MS; all authors revised the paper and approved the final version to be published.

## Funding

This work was funded by the “863” Key Project to JX (2014AA10A601) from the Chinese Ministry of Science and Technology (http://www.863.gov.cn/); the Shenzhen Peacock Plan (http://www.szsti.gov.cn/) (#: 20130415095710361, Recipient: ZKL); the CAAS Innovative Team Awards to ZL and JX teams (http://www.caas.net.cn/), and the Bill and Melinda Gates Foundation project (#OPP1130530) to ZL.

### Conflict of interest statement

The authors declare that the research was conducted in the absence of any commercial or financial relationships that could be construed as a potential conflict of interest.

## Supplementary material

Supplementary Figure S1 **Frequency of markers in different MAF classes of 22,488 SNP loci**.

Supplementary Table S1 **Origins and groups of 258 rice accessions**.

Supplementary Table S2 **Haplotype analysis of 19 candidate genes for seven important QTL regions**.

Supplementary Table S3 **Three accessions with wide GW and low chalkiness and their alleles at QTL for GW and chalkiness traits. Alleles in red and green colors represented the increased and reduced phenotypic values, respectively**.
