# Supplementary figures and images for: New Candidate Genes Affecting Rice Grain Appearance and Milling Quality Detected by Genome-Wide and Gene-Based Association Analyses
Source: Front Plant Sci. 2017 Jan 4;7:1998. doi: 10.3389/fpls.2016.01998 (PMC5209347; doi:10.3389/fpls.2016.01998)

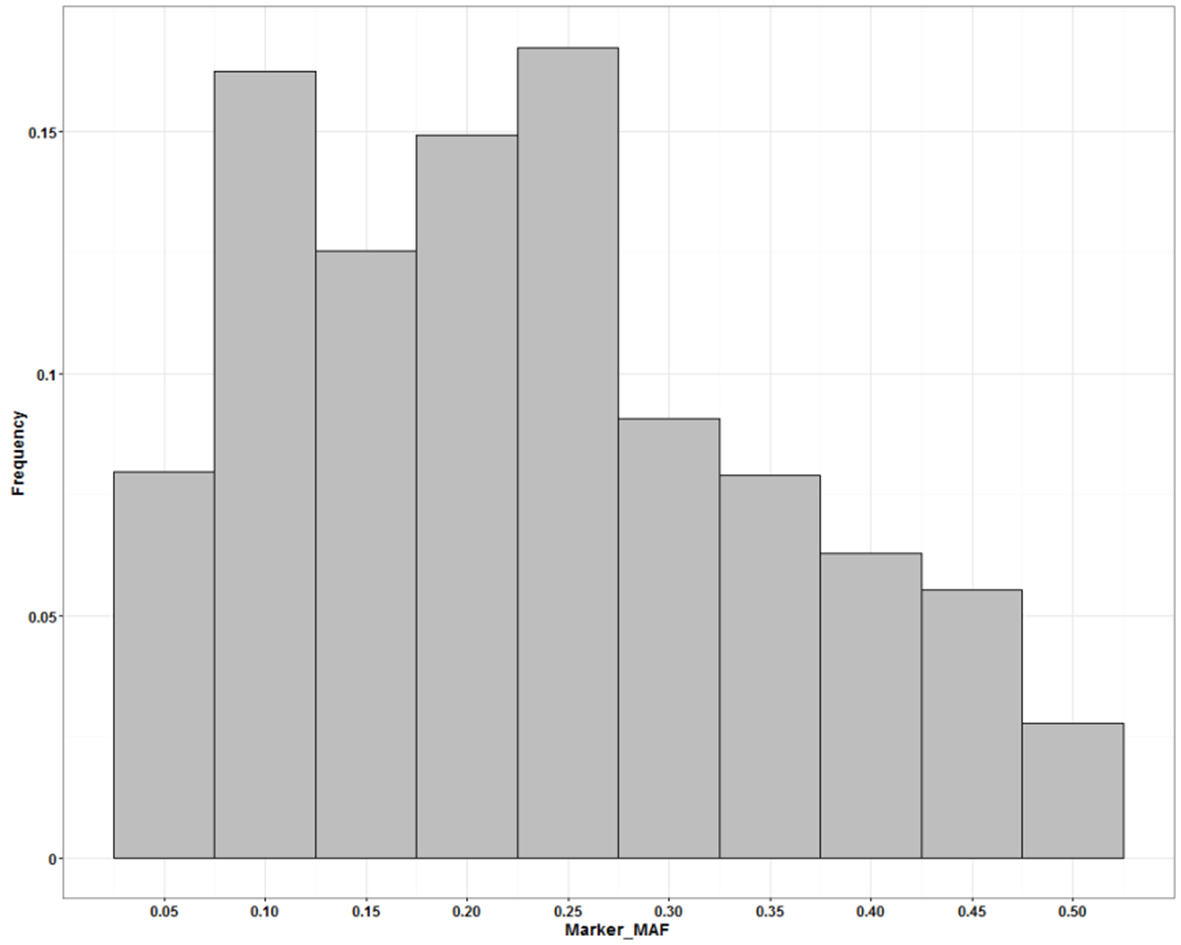

Supplement: Supplementary Figure S1 — Frequency of markers in different MAF classes of 22,488 SNP loci. [file Image1.TIF]
