# Supplementary material for: New Candidate Genes Affecting Rice Grain Appearance and Milling Quality Detected by Genome-Wide and Gene-Based Association Analyses
Source: Front Plant Sci. 2017 Jan 4;7:1998. doi: 10.3389/fpls.2016.01998 (PMC5209347; doi:10.3389/fpls.2016.01998)
Supplement: Supplementary Table S1 — Origins and groups of 258 rice accessions. [file Table1.DOCX]

# Supplementary Table S1 | Origins and groups of 258 rice accessions

| IRID No. | Origin | Group | IRID No. | Origin | Group |
| --- | --- | --- | --- | --- | --- |
| IRIS_313-11461 | India | *Aus/boro* | IRIS_313-7638 | Madagascar | *Indica* |
| IRIS_313-11491 | India | *Aus/boro* | IRIS_313-7641 | Indonesia | *Indica* |
| IRIS_313-8641 | Bangladesh | *Aus/boro* | IRIS_313-7650 | Madagascar | *Indica* |
| IRIS_313-11069 | Bangladesh | *Basmati/sadri* | IRIS_313-7651 | Madagascar | *Indica* |
| IRIS_313-11218 | Unknown | *Basmati/sadri* | IRIS_313-7665 | Colombia | *Indica* |
| IRIS_313-11297 | India | *Basmati/sadri* | IRIS_313-7698 | Philippines | *Indica* |
| IRIS_313-11824 | India | *Basmati/sadri* | IRIS_313-7720 | Madagascar | *Indica* |
| B051 | Egypt | *Indica* | IRIS_313-7758 | Madagascar | *Indica* |
| B085 | China | *Indica* | IRIS_313-7769 | Philippines | *Indica* |
| B238 | China | *Indica* | IRIS_313-7778 | Vietnam | *Indica* |
| B239 | China | *Indica* | IRIS_313-7792 | Madagascar | *Indica* |
| CX115 | China | *Indica* | IRIS_313-7808 | Senegal | *Indica* |
| CX123 | China | *Indica* | IRIS_313-7809 | Senegal | *Indica* |
| CX130 | India | *Indica* | IRIS_313-7815 | Senegal | *Indica* |
| CX131 | Myanmar | *Indica* | IRIS_313-7824 | Senegal | *Indica* |
| CX206 | Philippines | *Indica* | IRIS_313-7826 | Senegal | *Indica* |
| CX225 | Philippines | *Indica* | IRIS_313-7832 | Senegal | *Indica* |
| CX230 | China | *Indica* | IRIS_313-7863 | Unknown | *Indica* |
| CX233 | Philippines | *Indica* | IRIS_313-8063 | Argentina | *Indica* |
| CX237 | Philippines | *Indica* | IRIS_313-8147 | India | *Indica* |
| CX240 | Brazil | *Indica* | IRIS_313-8212 | Egypt | *Indica* |
| CX267 | India | *Indica* | IRIS_313-8216 | Greece | *Indica* |
| CX270 | Chinese Taipei | *Indica* | IRIS_313-8253 | Thailand | *Indica* |
| CX276 | Philippines | *Indica* | IRIS_313-8412 | China | *Indica* |
| CX303 | China | *Indica* | IRIS_313-8571 | Tanzania | *Indica* |
| CX304 | China | *Indica* | IRIS_313-8699 | Sri Lanka | *Indica* |
| CX318 | China | *Indica* | IRIS_313-8757 | India | *Indica* |
| CX343 | China | *Indica* | IRIS_313-8846 | Indonesia | *Indica* |
| CX366 | China | *Indica* | IRIS_313-8900 | India | *Indica* |
| CX375 | Africa | *Indica* | IRIS_313-8924 | India | *Indica* |
| CX377 | Africa | *Indica* | IRIS_313-8925 | Sri Lanka | *Indica* |
| CX378 | China | *Indica* | IRIS_313-8946 | India | *Indica* |
| CX393 | China | *Indica* | IRIS_313-9039 | Sri Lanka | *Indica* |
| CX394 | China | *Indica* | IRIS_313-9119 | Thailand | *Indica* |
| CX42 | Philippines | *Indica* | IRIS_313-9317 | Vietnam | *Indica* |
| CX50 | China | *Indica* | IRIS_313-9325 | Bangladesh | *Indica* |
| CX51 | China | *Indica* | IRIS_313-9348 | India | *Indica* |
| CX61 | India | *Indica* | IRIS_313-9472 | Sri Lanka | *Indica* |
| CX80 | Vietnam | *Indica* | IRIS_313-9484 | India | *Indica* |
| CX82 | Vietnam | *Indica* | IRIS_313-9547 | India | *Indica* |
| CX93 | india | *Indica* | IRIS_313-9560 | India | *Indica* |
| IRIS_313-10001 | Chinese Taipei | *Indica* | IRIS_313-9572 | Bhutan | *Indica* |
| IRIS_313-10002 | Sri Lanka | *Indica* | IRIS_313-9602 | Thailand | *Indica* |
| IRIS_313-10034 | Niger | *Indica* | IRIS_313-9611 | India | *Indica* |
| IRIS_313-10040 | Korea, Republic Of | *Indica* | IRIS_313-9706 | Chinese Taipei | *Indica* |
| IRIS_313-10041 | Madagascar | *Indica* | IRIS_313-9730 | China | *Indica* |
| IRIS_313-10045 | Gambia | *Indica* | IRIS_313-9732 | Madagascar | *Indica* |
| IRIS_313-10050 | Madagascar | *Indica* | IRIS_313-9740 | Madagascar | *Indica* |
| IRIS_313-10113 | Mozambique | *Indica* | IRIS_313-9791 | Malawi | *Indica* |
| IRIS_313-10129 | China | *Indica* | IRIS_313-9822 | Venezuela | *Indica* |
| IRIS_313-10134 | Thailand | *Indica* | IRIS_313-9922 | Korea, Republic Of | *Indica* |
| IRIS_313-10151 | Thailand | *Indica* | IRIS_313-9924 | Korea, Republic Of | *Indica* |
| IRIS_313-10211 | China | *Indica* | CX342 | China | *Intermediate type* |
| IRIS_313-10224 | China | *Indica* | CX347 | China | *Intermediate type* |
| IRIS_313-10268 | Venezuela | *Indica* | IRIS_313-11290 | India | *Intermediate type* |
| IRIS_313-10285 | Peru | *Indica* | IRIS_313-11627 | Nepal | *Intermediate type* |
| IRIS_313-10333 | Indonesia | *Indica* | IRIS_313-7797 | Philippines | *Intermediate type* |
| IRIS_313-10337 | Indonesia | *Indica* | IRIS_313-8409 | Vietnam | *Intermediate type* |
| IRIS_313-10341 | Bangladesh | *Indica* | IRIS_313-9503 | Philippines | *Intermediate type* |
| IRIS_313-10349 | India | *Indica* | CX241 | Philippines | *japonica* |
| IRIS_313-10352 | Colombia | *Indica* | CX277 | China | *japonica* |
| IRIS_313-10360 | Philippines | *Indica* | CX351 | China | *japonica* |
| IRIS_313-10375 | Philippines | *Indica* | IRIS_313-10067 | Korea, Republic Of | *Japonica* |
| IRIS_313-10399 | Colombia | *Indica* | IRIS_313-10077 | Japan | *Japonica* |
| IRIS_313-10417 | India | *Indica* | IRIS_313-10293 | Cuba | *Japonica* |
| IRIS_313-10497 | China | *Indica* | IRIS_313-10423 | Myanmar | *Japonica* |
| IRIS_313-10515 | Chinese Taipei | *Indica* | IRIS_313-11789 | Madagascar | *Japonica* |
| IRIS_313-10576 | Sierra Leone | *Indica* | IRIS_313-7993 | Madagascar | *Japonica* |
| IRIS_313-10640 | Japan | *Indica* | IRIS_313-8003 | Philippines | *Japonica* |
| IRIS_313-10666 | India | *Indica* | IRIS_313-8061 | Argentina | *Japonica* |
| IRIS_313-10683 | Unknown | *Indica* | IRIS_313-8164 | Australia | *Japonica* |
| IRIS_313-10733 | Nepal | *Indica* | IRIS_313-8193 | United States | *Japonica* |
| IRIS_313-10863 | India | *Indica* | IRIS_313-8303 | India | *Japonica* |
| IRIS_313-10904 | Cambodia | *Indica* | B002 | China | *Temperate japonica* |
| IRIS_313-10971 | Bangladesh | *Indica* | B023 | North Korea | *Temperate japonica* |
| IRIS_313-10996 | Indonesia | *Indica* | CX116 | North Korea | *Temperate japonica* |
| IRIS_313-11120 | Bhutan | *Indica* | IRIS_313-10014 | Italy | *Temperate japonica* |
| IRIS_313-11140 | Myanmar | *Indica* | IRIS_313-10379 | Philippines | *Temperate japonica* |
| IRIS_313-11142 | Myanmar | *Indica* | IRIS_313-10430 | Japan | *Temperate japonica* |
| IRIS_313-11144 | Myanmar | *Indica* | IRIS_313-10564 | Japan | *Temperate japonica* |
| IRIS_313-11147 | Myanmar | *Indica* | IRIS_313-10567 | Japan | *Temperate japonica* |
| IRIS_313-11221 | Bangladesh | *Indica* | IRIS_313-10967 | Brazil | *Temperate japonica* |
| IRIS_313-11224 | Bangladesh | *Indica* | IRIS_313-11467 | Philippines | *Temperate japonica* |
| IRIS_313-11261 | India | *Indica* | IRIS_313-11478 | India | *Temperate japonica* |
| IRIS_313-11262 | India | *Indica* | IRIS_313-11487 | Bangladesh | *Temperate japonica* |
| IRIS_313-11266 | India | *Indica* | IRIS_313-11573 | China | *Temperate japonica* |
| IRIS_313-11287 | India | *Indica* | IRIS_313-11577 | China | *Temperate japonica* |
| IRIS_313-11296 | India | *Indica* | IRIS_313-11652 | China | *Temperate japonica* |
| IRIS_313-11307 | India | *Indica* | IRIS_313-11802 | China | *Temperate japonica* |
| IRIS_313-11338 | Philippines | *Indica* | IRIS_313-11828 | India | *Temperate japonica* |
| IRIS_313-11347 | Philippines | *Indica* | IRIS_313-8025 | Italy | *Temperate japonica* |
| IRIS_313-11402 | Bangladesh | *Indica* | IRIS_313-8046 | Italy | *Temperate japonica* |
| IRIS_313-11418 | India | *Indica* | IRIS_313-8067 | Italy | *Temperate japonica* |
| IRIS_313-11549 | Myanmar | *Indica* | IRIS_313-8068 | Italy | *Temperate japonica* |
| IRIS_313-11571 | China | *Indica* | IRIS_313-8074 | Australia | *Temperate japonica* |
| IRIS_313-11599 | India | *Indica* | IRIS_313-8087 | Spain | *Temperate japonica* |
| IRIS_313-11607 | India | *Indica* | IRIS_313-8090 | Spain | *Temperate japonica* |
| IRIS_313-11621 | India | *Indica* | IRIS_313-8132 | Portugal | *Temperate japonica* |
| IRIS_313-11642 | India | *Indica* | IRIS_313-8161 | Brazil | *Temperate japonica* |
| IRIS_313-11647 | India | *Indica* | IRIS_313-8173 | United States | *Temperate japonica* |
| IRIS_313-11648 | India | *Indica* | IRIS_313-8205 | Italy | *Temperate japonica* |
| IRIS_313-11692 | Chinese Taipei | *Indica* | IRIS_313-9346 | Chinese Taipei | *Temperate japonica* |
| IRIS_313-11698 | Chinese Taipei | *Indica* | IRIS_313-9701 | Chinese Taipei | *Temperate japonica* |
| IRIS_313-11731 | China | *Indica* | IRIS_313-9702 | Chinese Taipei | *Temperate japonica* |
| IRIS_313-11740 | Ghana | *Indica* | IRIS_313-9724 | China | *Temperate japonica* |
| IRIS_313-11772 | Madagascar | *Indica* | B043 | Australia | *Tropical japonica* |
| IRIS_313-11779 | Tanzania | *Indica* | B053 | Australia | *Tropical japonica* |
| IRIS_313-11782 | Zambia | *Indica* | CX220 | Brazil | *Tropical japonica* |
| IRIS_313-11806 | China | *Indica* | IRIS_313-10190 | China | *Tropical japonica* |
| IRIS_313-11812 | Kenya | *Indica* | IRIS_313-10314 | Ecuador | *Tropical japonica* |
| IRIS_313-11821 | India | *Indica* | IRIS_313-11525 | Guinea-Bissau | *Tropical japonica* |
| IRIS_313-11853 | China | *Indica* | IRIS_313-11754 | Madagascar | *Tropical japonica* |
| IRIS_313-11854 | China | *Indica* | IRIS_313-11796 | China | *Tropical japonica* |
| IRIS_313-11859 | China | *Indica* | IRIS_313-11844 | Thailand | *Tropical japonica* |
| IRIS_313-11882 | China | *Indica* | IRIS_313-11922 | Thailand | *Tropical japonica* |
| IRIS_313-11885 | China | *Indica* | IRIS_313-7699 | Philippines | *Tropical japonica* |
| IRIS_313-11887 | Philippines | *Indica* | IRIS_313-7866 | Colombia | *Tropical japonica* |
| IRIS_313-11910 | China | *Indica* | IRIS_313-7868 | Colombia | *Tropical japonica* |
| IRIS_313-11911 | China | *Indica* | IRIS_313-7933 | Nepal | *Tropical japonica* |
| IRIS_313-11918 | India | *Indica* | IRIS_313-8010 | Philippines | *Tropical japonica* |
| IRIS_313-11932 | Kenya | *Indica* | IRIS_313-8075 | Australia | *Tropical japonica* |
| IRIS_313-11939 | Burkina Fasso | *Indica* | IRIS_313-8134 | Portugal | *Tropical japonica* |
| IRIS_313-11954 | China | *Indica* | IRIS_313-8140 | China | *Tropical japonica* |
| IRIS_313-12012 | China | *Indica* | IRIS_313-8213 | France | *Tropical japonica* |
| IRIS_313-12013 | India | *Indica* | IRIS_313-8285 | United States | *Tropical japonica* |
| IRIS_313-12083 | Madagascar | *Indica* | IRIS_313-8976 | United States | *Tropical japonica* |
| IRIS_313-12288 | Myanmar | *Indica* | IRIS_313-9120 | India | *Tropical japonica* |
| IRIS_313-12290 | Myanmar | *Indica* | IRIS_313-9366 | United States | *Tropical japonica* |
| IRIS_313-12325 | Cameroon | *Indica* | IRIS_313-9771 | Austria | *Tropical japonica* |
