# Supplementary material for: New Candidate Genes Affecting Rice Grain Appearance and Milling Quality Detected by Genome-Wide and Gene-Based Association Analyses
Source: Front Plant Sci. 2017 Jan 4;7:1998. doi: 10.3389/fpls.2016.01998 (PMC5209347; doi:10.3389/fpls.2016.01998)
Supplement: Supplementary Table S3 — Three accessions with wide GW and low chalkiness and their alleles at QTL for GW and chalkiness traits. Alleles in red and green colors represented the increased and reduced phenotypic values, respectively. [file Table3.DOCX]

Supplementary Table S3 | Three accessions with wide GW and low chalkiness and their alleles at QTL for GW and chalkiness traits

| Trait /QTL ^a^ | Accession and alleles at QTL ^b^ | | |
| --- | --- | --- | --- |
|  | IRIS_313.10430 | IRIS_313.8087 | IRIS_313.8164 |
| GW in SY | 2.7 | 2.7 | 2.6 |
| PGWC in SY | 2.6 | 3.0 | 5.3 |
| DEC in SY | 0.6 | 0.6 | 1.4 |
| Tr in SY | 1 | 1 | 1 |
| GW in SZ | 2.8 | 2.6 | 2.8 |
| PGWC in SZ | 9.4 | 10.1 | 14.6 |
| DEC in SZ | 2.1 | 3.0 | 4.0 |
| Tr at SZ | 2 | 2 | 2 |
| *qGW3.1* | C | C | C |
| *qGW4.1* | T | T | T |
| *qGW4.2* | G | G | G |
| *qGW6* | C | C | C |
| *qGW9* | A | A | A |
| *qGW1.1* | A | A | C |
| *qGW1.2* | A | A | G |
| *qGW7.4* | G | G | T |
| *qGW11* | C | C | A |
| *qGW3.2* | A | A | A |
| *qGW7.1* | G | G | G |
| *qGW7.2* | C | C | C |
| *qGW7.3* | T | T | T |
| *qGW8.1* | A | A | A |
| *qGW10.2* | C | C | T |
| *qGW5* (*qPGWC5*) | G | G | G |
| *qGW8.2* (*qDEC8, qPGWC8*) | A | A | A |
| *qGW10.1*(*qPGWC10*) | C | C | C |
| *qDEC1* | A | A | A |
| *qDEC3* | C | C | C |
| *qDEC7 (qTr7.2)* | C | C | C |
| *qPGWC3* | T | T | T |
| *qTr1* | A | A | A |
| *qTr4* | C | C | C |
| *qTr7.1* | A | A | A |
| *qTr2* | T | T | T |
| *qTr7.3* | C | C | C |

^a^ SY: Sanya. SZ: Shenzhen. GW: grain width. PGWC: percentage of grains with chalkiness. DEC: degree of endosperm chalkiness. Tr: transparency.

^b^ Alleles in red and green colors represent the increased and reduced phenotypic values, respectively.
